# Nighttime Outdoor Artificial Light and Risk of Age-Related Macular Degeneration

**DOI:** 10.1001/jamanetworkopen.2023.51650

**Published:** 2024-01-16

**Authors:** Su Hwan Kim, Young Kook Kim, Young In Shin, Goneui Kang, Seong Pyo Kim, Hajoung Lee, In Hwan Hong, In Boem Chang, Soon-Beom Hong, Hyung-Jin Yoon, Ahnul Ha

**Affiliations:** 1 Biomedical Research Institute, Seoul National University Hospital (SNUH), Seoul, Korea; 2 Department of Ophthalmology, SNUH, Seoul, Korea; 3 Department of Ophthalmology, Seoul National University College of Medicine, Seoul, Korea; 4 EyeLight Data Science Laboratory, Seoul National University College of Medicine, Seoul, Korea; 5 Interdisciplinary Program of Medical Informatics, Seoul National University College of Medicine, Seoul, Korea; 6 Department of Ophthalmology, Dongtan Sacred Heart Hospital, Hwaseong, Korea; 7 Department of Ophthalmology, Hallym University Medical Center, Hwaseong, Korea; 8 Seoul ON Eye Clinic, Seoul, Korea; 9 Department of Psychiatry, Seoul National University College of Medicine, Seoul, Korea; 10 Department of Psychiatry, SNUH, Seoul, Korea; 11 Institute of Human Behavioral Medicine, SNU Medical Research Center, Seoul, Korea; 12 Medical Bigdata Research Center, SNU College of Medicine, Seoul, Korea; 13 Department of Ophthalmology, Jeju National University Hospital, Jeju, Korea; 14 Department of Ophthalmology, Jeju National University College of Medicine, Jeju, Korea

## Abstract

**Importance:**

Light pollution’s impact on human health is increasingly recognized, but its link to exudative age-related macular degeneration (EAMD) remains unclear.

**Objective:**

To investigate the association between exposure to outdoor artificial light at night (OALAN) and the risk of incident EAMD.

**Design, Setting, and Participants:**

In this nationwide population-based case-control study, all individuals 50 years or older with newly diagnosed EAMD between January 1, 2010, and December 31, 2011, were identified with reference to the Korean National Health Insurance Service registration program database for rare and intractable diseases. Birth year– and sex-matched controls (with no EAMD diagnosis until 2020) were selected at a 1:30 ratio. Data were acquired from May 1 to December 31, 2021, and analyzed from June 1 to November 30, 2022.

**Exposures:**

Mean levels of OALAN at participants’ residential addresses during 2008 and 2009 were estimated using time-varying satellite data for a composite view of persistent nighttime illumination at an approximate scale of 1 km^2^.

**Main Outcomes and Measures:**

The hazard ratios (HRs) and 95% CIs of the association between residential OALAN and risk of incident EAMD were determined based on maximum likelihood estimation after adjusting for sociodemographic characteristics, comorbidities, and area-level risk factors (ie, nighttime traffic noise and particulate matter of aerodynamic diameter ≤10 μm in each participant’s administrative district of residence).

**Results:**

A total of 126 418 participants were included in the analysis (mean [SD] age, 66.0 [7.9] years; 78 244 men [61.9%]). Of these, 4078 were patients with newly diagnosed EAMD and 122 340 were EAMD-free matched controls. In fully adjusted models, an IQR (55.8 nW/cm^2^/sr) increase in OALAN level was associated with an HR of 1.67 (95% CI, 1.56-1.78) for incident EAMD. The exposure-response curve demonstrated a nonlinear, concave upward slope becoming more pronounced at higher levels of light exposure (ie, at approximately 110 nW/cm^2^/sr). In a subgroup analysis, an IQR increase in OALAN was associated with increased risk of incident EAMD in urban areas (HR, 1.46 [95% CI, 1.33-1.61]) but not in rural areas (HR, 1.01 [95% CI, 0.84-1.22]).

**Conclusions and Relevance:**

In this nationwide population-based case-control study, higher levels of residential OALAN were associated with an increased risk of incident EAMD. Future studies with more detailed information on exposure, individual adaptive behaviors, and potential mediators are warranted.

## Key Points

**Question:**

Is exposure to outdoor artificial light at night (OALAN) associated with the occurrence of exudative age-related macular degeneration (EAMD)?

**Findings:**

In this population-based case-control study of 4078 patients newly diagnosed with EAMD and 122 340 individuals without EAMD, a positive association between higher levels of residential OALAN and the risk of developing incident EAMD was observed. The association between exposure to light and EAMD followed a nonlinear pattern, with a concave upward slope that became more pronounced at higher levels of light exposure.

**Meaning:**

These findings suggest that OALAN may be a risk factor for EAMD.

## Teaser

This case-control study investigates the association between residential outdoor artificial light at night and the risk of incident exudative age-related macular degeneration using nationwide population-based data in South Korea.

## Introduction

Age-related macular degeneration (AMD) is a chronic disease that affects the macular region of the retina, causing progressive loss of central vision.^1^ It is the leading cause of irreversible blindness worldwide, and its prevalence is expected to rise in line with the exponential increase in the aging population.^2^ Various genetic as well as nongenetic risk factors for AMD, a multifactorial disorder with strong genetic components, have been widely investigated.^3^ The most consistent nongenetic risk factors for development and progression of AMD, apart from age, have been cigarette smoking and low dietary intake of antioxidants.^1,4^ While these traditional risk factors continue to play a substantial role, environmental factors associated with AMD risk and their significance are attracting growing research interest.^5,6,7^

One of the most widespread yet often overlooked environmental hazards resulting from human activity is light pollution.^8^ This is characterized by disturbance of the nocturnal environment due to light emitted from anthropogenic sources, and its negative effects on human health recently have become a pressing concern.^9^ The potential detrimental health impacts of light pollution have expanded beyond sleep disorders^10,11,12^ to include obesity,^13,14^ cardiovascular diseases,^15,16,17^ cancers,^18,19,20,21^ and mental disorders.^22,23^ The primary mechanism through which these effects occur is believed to be the disruption of circadian rhythms and subsequent impaired hormone secretion, particularly those associated with sleep disturbances.^24^

Artificial light at night (ALAN) specifically refers to the alteration of natural light levels at night caused by human-made light sources.^9^ It has been widely acknowledged that ALAN has a substantial impact on the retina and optic nerve, potentially resulting in both indirect and direct damage to ocular tissue.^25^ Our objective was to investigate the association between residential outdoor artificial light at night (OALAN) and risk of incident exudative AMD (EAMD) using nationwide population-based data in South Korea.

## Methods

In this case-control study, we accessed the Korean National Health Insurance Service (NHIS) database between 2008 and 2020. The NHIS provides mandatory health insurance coverage for 99.8% of the South Korean population. The NHIS database includes demographic information as well as medical records data related to diagnoses, dates of medical visits, examinations, prescriptions, and procedures. Diagnoses are recorded in accordance with the *International Statistical Classification of Diseases and Related Health Problems, Tenth Revision*. Additionally, we used data from the National Health Screening database, which is linked with the NHIS database, as reported in a previous study.^26^ All NHIS enrollees 18 years or older are required to undergo a biennial health screening, provided free of charge, that includes anthropometric measurements, assessment of blood pressure, laboratory tests, and a self-reported questionnaire on health behaviors such as smoking, alcohol consumption, and physical activity.

This study obtained ethical clearance from the Institutional Review Board of Seoul National University Hospital, Seoul, Korea, and the NHIS Deliberative Committee granted conditional approval for the use of the Health Insurance Review and Assessment database. The institutional review board of Seoul National University Hospital waived the requirement for informed consent, as the data had been anonymized and accessed at a closed-network onsite location. This study was performed in accordance with the principles of the Declaration of Helsinki^27^ and designed according to the Strengthening the Reporting of Observational Studies in Epidemiology (STROBE) reporting guideline.

### Study Population and Outcome Ascertainment

Taking into account the typical age of onset,^3^ we included individuals 50 years or older who had been newly diagnosed with EAMD between January 1, 2010, and December 31, 2011. The incorporation of patients diagnosed in 2010 and 2011 aimed to ensure a follow-up period of at least 10 years by 2020, the year when the data were acquired. Given that patients with EAMD typically visit the hospital at least once a year, a washout period of 2 years (2008-2009) was implemented to thoroughly exclude individuals with a prior diagnosis.^28^ We also selected 1:30 birth year– and sex-matched controls who had not been diagnosed with EAMD until 2020. The exposure assessment time frame in the control group was the same as that in the case group.

The identification of patients with EAMD and the controls was based on the NHIS registration program database of rare and intractable diseases (code V201), which includes cases (1) confirmed by an ophthalmologist to be EAMD for copayment reduction purposes^28,29^ and (2) undergoing strict Health Insurance Review and Assessment expert panel reviews of case eligibility at initial registration and verification of EAMD diagnoses’ accuracy and reliability. Cases with missing data in any of the covariates were excluded from the final analysis.

### Assessment of OALAN

The source of the OALAN data was the US Air Force Defense Meteorological Satellite Program Operational Linescan System (DMSP-OLS).^30^ The DMSP-OLS data provide a 30 × 30 arc-second gridded nocturnal luminosity equivalent to approximately 1 km^2^.^31^ The National Geophysical Data Center corrects for the saturation of DMSP-OLS data by merging low, medium, and high fixed-gain image levels and releases global data sets of radiance-calibrated light at night.^32^ To ensure comparability across years and satellites, we used interannual calibration coefficients provided by the US National Oceanic and Atmospheric Administration to derive exposure estimates.^32^

**Figure 1.**
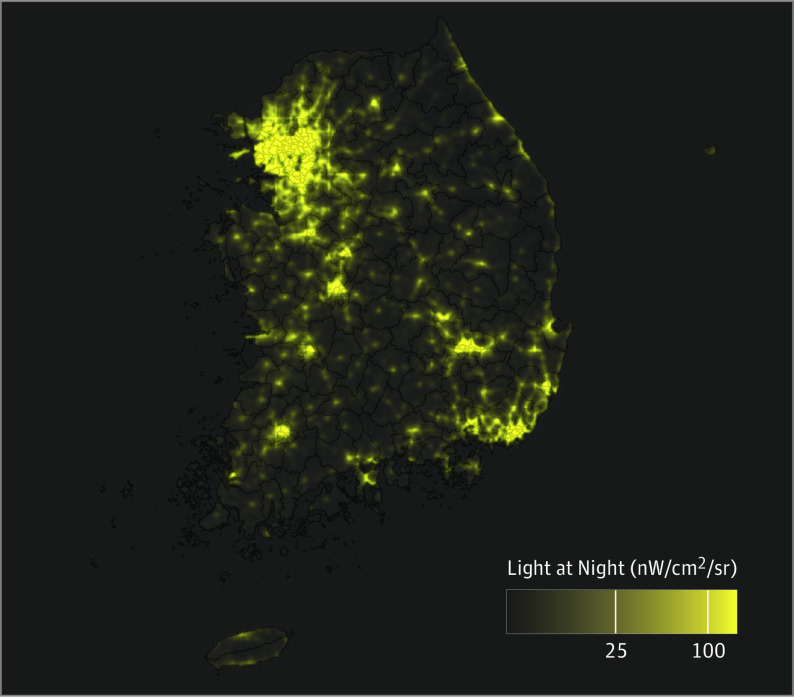
Map of South Korea Showing 2008-2009 Outdoor Artificial Light at Night Data are shown in nanowatts per centimeter squared per steradian (nW/cm^2^/sr). The data were obtained by the Defense Meteorological Satellite Program’s Operational Linescan System. The solid lines delineate the 250 districts of South Korea.

We calculated an objective measure of mean OALAN, expressed in units of radiance as nanowatts per centimeter squared per steradian, for each geocoded address for the years 2008 to 2009 using the National Geophysical Data Center’s latest radiance-calibrated light-at-night data (Figure 1). Such data have been proposed as a reliable proxy measure for relative levels of nighttime illumination at ground level and have been used recently to assess the effects of light pollution on various comorbidities.^15,21,33^

### Statistical Analysis

Data were acquired from May 1 to December 31, 2021, and analyzed from June 1 to November 30, 2022. We used Cox proportional hazards regression to estimate the association between OALAN and the risk of incident EAMD. We first examined the association between OALAN and incident EAMD using a minimally adjusted model, including age and sex. We next used a fully adjusted model that additionally included adjustment for the following factors that are potentially associated with risk of EAMD: body mass index (BMI; calculated as weight in kilograms divided by height in meters squared), smoking status, physical activity status, alcohol consumption, income level, and comorbidities, including hypertension, type 2 diabetes, dyslipidemia, chronic kidney disease, chronic obstructive pulmonary disease, and cancer (eTable 1 in Supplement 1). Additionally, given the close association between OALAN and urban structure, we conducted adjustments for residential area (urban or rural), nighttime noise, and air pollution, specifically particulate matter less than or equal to 10 μm in aerodynamic diameter (PM_10_) at the residential address (eMethods in Supplement 1). We assessed the proportional hazards assumption to ascertain the presence of time-varying effects on the covariate coefficients (eTable 2 in Supplement 1). Also, we conducted a multicollinearity analysis among the independent variables (eTable 3 in Supplement 1).

We examined the association between OALAN and EAMD risk using 3 different approaches: hazard ratio (HR) per IQR increase (calculated based on the entire study population) in continuous OALAN with the linearity assumption, HRs for each of the upper 3 quartiles relative to the lowest OALAN quartile, and an exposure-response HR curve describing the nonlinear relationship by penalized smoothing spline with 4 *df*. For the quartile analysis, we conducted a linearity test for trends across the quartiles, where the medians of each quartile were used as exploratory variables and the corresponding HRs were used as continuous dependent variables in the model.

We performed a sensitivity analysis by repeating the main analyses with additional adjustment for depression and sleep disorders, which are known to affect sleep quality. Furthermore, we explored the possibility of effect modification by various factors, including age at baseline, sex, BMI, smoking status, physical activity status, alcohol consumption, residential area (urban or rural), income level, and history of underlying diseases, to determine whether the association between OALAN and EAMD varied among different subpopulations. We tested the homogeneity across stratum-specific HRs using the interaction between continuous OALAN and each potential modifier.

Two-sided *P* < .05 was considered statistically significant. Data management and analysis were conducted using SAS, version 9.4 (SAS Institute Inc), and plots were generated using R software, version 4.0.0 (R Project for Statistical Computing).

## Results

Initially, this study considered a total of 13 364 patients with newly diagnosed EAMD (mean [SD] age, 68.8 [8.9] years; 7818 [58.5%] men and 5546 women [41.5%]) and 2 063 554 EAMD-free individuals (mean [SD] age, 59.2 [14.9] years; 907 964 [44.0%] men and 1 155 590 [56.0%] women). After applying the exclusion criteria and performing matching, 4078 patients with newly diagnosed EAMD and 122 340 EAMD-free individuals were included in the study for a total of 126 418 participants (78 244 men [61.9%] and 48 174 women [38.1%]; mean [SD] age, 66.0 [7.9] years) (eFigure 1 in Supplement 1). Summary statistics for the baseline characteristics of the participants are shown in Table 1. We found that participants in the higher quartile of OALAN tended to reside in areas with higher levels of PM_10_ and nighttime traffic noise. Conversely, those in the lower quartiles of OALAN reported lower levels of physical activity, as shown in eTable 4 in Supplement 1.

**Table 1.** Baseline Characteristics of Participants^a^

Characteristic	Overall (N = 126 418)^b^	Urban area (n = 73 551)	Rural area (n = 48 972)	*P* value
Light at night, mean (SD), nW/cm^2^/sr	45.0 (35.6)	61.2 (34.8)	20.6 (18.8)	<.001
Participants in each quartile				
Quartile 1	30 937 (25.2)	3893 (5.3)	27 044 (55.2)	<.001
Quartile 2	30 049 (24.5)	18 521 (25.2)	11 528 (23.5)	
Quartile 3	31 379 (25.6)	20 984 (28.5)	10 395 (21.2)	
Quartile 4	30 158 (24.6)	30 153 (41.0)	5 (0.01)	
Area-level factors, mean (SD)				
PM_10_, mg/m^3^	52.9 (5.0)	54.2 (5.1)	52.0 (4.8)	<.001
Nighttime traffic noise, dB	55.4 (3.4)	56.3 (3.4)	53.9 (2.8)	<.001
Age, y				
Mean (SD)	66.0 (7.9)	63.7 (8.1)	69.4 (6.4)	<.001
<70	75 547 (59.8)	52 580 (71.5)	20 854 (42.6)	<.001
≥70	50 871 (40.2)	20 971 (28.5)	28 118 (57.4)	
Sex				
Men	78 244 (61.9)	49 415 (67.2)	26 744 (54.6)	<.001
Women	48 174 (38.1)	24 136 (32.8)	22 228 (45.4)	
BMI, mean (SD)	24.0 (3.0)	24.0 (2.9)	24.1 (3.1)	<.001
Comorbidities				
Hypertension	89 485 (70.8)	49 347 (67.1)	37 292 (76.1)	<.001
Diabetes	15 376 (12.2)	9002 (12.2)	5964 (12.2)	.38
Dyslipidemia	16 042 (12.7)	9234 (12.6)	6262 (12.8)	.12
COPD	20 913 (16.5)	11 369 (15.5)	8887 (18.1)	<.001
CKD	2610 (2.1)	1320 (1.8)	1205 (2.5)	<.001
Cancer	5380 (4.3)	3013 (4.1)	2201 (5.5)	<.001
Smoking status				
Never	82 372 (65.2)	45 107 (61.3)	34 552 (70.6)	<.001
Former	22 044 (17.4)	14 171 (19.3)	7258 (14.8)	
Current	22 002 (17.4)	14 273 (19.4)	7162 (14.6)	
Alcohol consumption				
None	80 899 (64.0)	43 960 (59.8)	34 238 (69.9)	<.001
Mild	21 367 (16.9)	13 704 (18.6)	7072 (14.4)	
Moderate	10 835 (8.6)	7337 (10.0)	3231 (6.6)	
Heavy	8517 (6.7)	5577 (7.6)	2724 (5.6)	
Extremely heavy	4800 (3.8)	2973 (4.0)	1707 (3.5)	
Exercise (physical activity)				
Light	55 175 (43.6)	29 168 (39.7)	24 294 (49.6)	<.001
Moderate	52 475 (41.5)	33 029 (44.9)	17 825 (36.4)	
Vigorous	18 768 (14.8)	11 354 (15.4)	6853 (14.0)	
Household income				
Quartile 1	23 158 (18.3)	13 759 (18.7)	8703 (17.8)	<.001
Quartile 2	20 367 (16.1)	12 284 (16.7)	7512 (15.3)	
Quartile 3	29 606 (23.4)	17 545 (23.9)	11 115 (22.7)	
Quartile 4	53 287 (42.2)	29 963 (40.7)	21 642 (44.2)	
Incident EAMD	4078 (3.2)	2755 (3.7)	1210 (2.5)	<.001

Abbreviations: BMI, body mass index (calculated as weight in kilograms divided by height in meters squared); CKD, chronic kidney disease; COPD, chronic obstructive pulmonary disease; EAMD, exudative age-related macular degeneration; PM_10_, particulate matter with aerodynamic diameter of 10 μm or less.

^a^ Continuous variables are presented as mean (SD) where indicated; categorical variables are presented as No. (%) of participants. Percentages have been rounded and may not total 100.

^b^ The overall total exceeds the sum of urban and rural participants because 3895 participants changed their place of residence during the study period.

### OALAN and EAMD Risk

**Figure 2.**
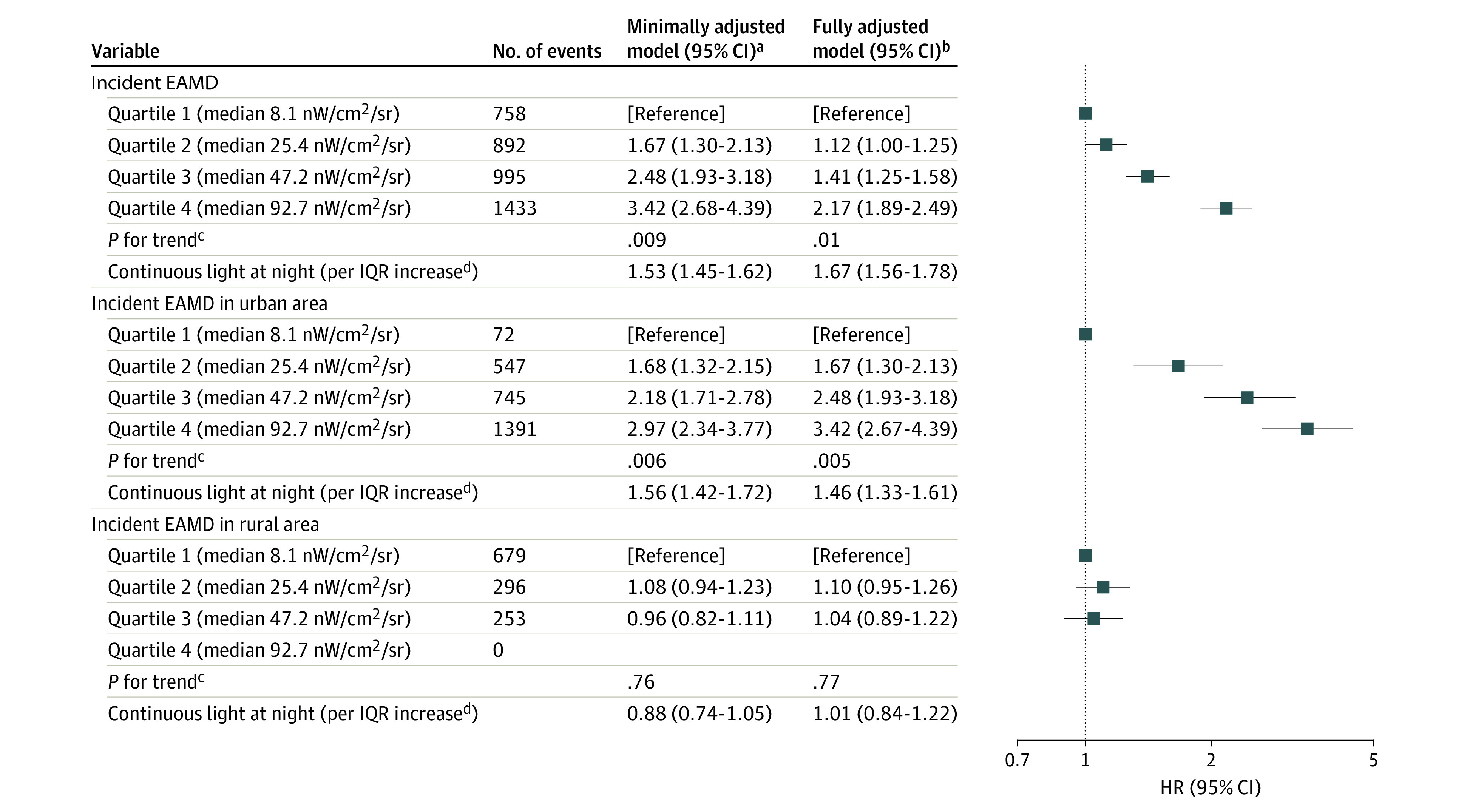
Association Between Outdoor Artificial Light at Night and Risk of Exudative Age-Related Macular Degeneration (EAMD) HR indicates hazard ratio. ^a^Models included age and sex. ^b^Models were additionally adjusted for baseline body mass index; alcohol consumption; exercise status; income level; comorbidities, including hypertension, type 2 diabetes, dyslipidemia, chronic kidney disease, chronic obstructive pulmonary disease, and cancer; fine particulate matter less than or equal to 10 μm in aerodynamic diameter (PM_10_); and nighttime traffic noise at the residential address. In the comprehensive model, residential area was also incorporated as a covariate. The analyses considered the interaction between residential areas and outdoor artificial light at night. ^c^Test for trend is based on the median value for each quartile. ^d^An IQR increase in outdoor light at night at the residential address is 55.8 nW/cm^2^/sr.

Higher OALAN at the residence was associated with a higher risk of incident EAMD (Figure 2). In fully adjusted models, the HR in the highest quartile was 2.17 (95% CI, 1.89-2.49) for incident EAMD. Even the second-lowest quartile had a significant risk of EAMD (HR, 1.12 [95% CI, 1.00-1.25]) relative to the lowest quartile, and the risk increased progressively with each higher quartile (*P* = .01). An IQR (55.8 nW/cm^2^/sr) increase in OALAN was associated with an HR of 1.67 (95% CI, 1.56-1.78) for incident EAMD. The detailed results for both minimally and fully adjusted Cox proportional hazards regression models for each analysis can be found in eTables 5 and 6 in Supplement 1.

**Figure 3.**
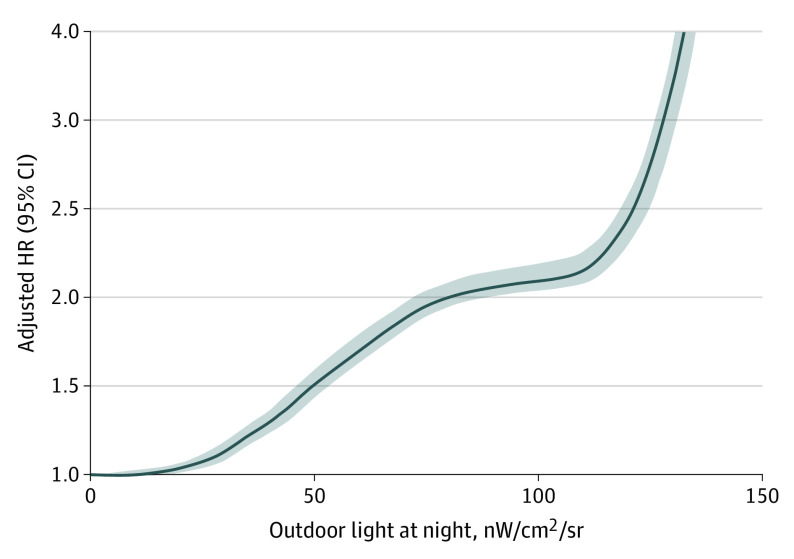
Exposure-Response Curve for Association Between Outdoor Artificial Light at Night (OALAN) at Residential Address and Risk of Incident Exudative Age-Related Macular Degeneration We fitted OALAN as a smooth term using a penalized smoothing spline with 4 *df*. The shaded area represents 95% CIs. HR indicates hazard ratio.

We examined the shape of the exposure-response curve for the association between OALAN and EAMD risk (Figure 3). The curve demonstrates a nonlinear, concave upward slope that becomes more pronounced at higher levels of OALAN (ie, approximately 110 nW/cm^2^/sr) (eFigure 2 in Supplement 1).

### Urban-Rural Differences in OALAN and EAMD Risk

A total of 122 523 participants who had not relocated between urban and rural areas during the study period were included in the subgroup analysis. Of these, 73 551 participants (60.0%) resided in urban areas, and 2755 of them (3.7%) experienced incident EAMD in 2010 to 2011 (Table 1). Of the 48 972 participants in rural areas, 1210 (2.5%) experienced EAMD during the same period. The mean (SD) OALAN in urban areas was 61.2 (34.8) nW/cm^2^/sr, while that in rural areas was 20.6 (18.8) nW/cm^2^/sr.

In the analysis of the interaction between residential areas and OALAN, a significantly higher risk of incident EAMD was observed in urban areas (*P* = .005) (Figure 2). In fully adjusted models, the HR in the highest quartile was 3.42 (95% CI, 2.67-4.39) for incident EAMD. An IQR (55.8 nW/cm^2^/sr) increase in OALAN was associated with an HR of 1.46 (95% CI, 1.33-1.61) for incident EAMD. Similar results were found when analyzing only the 3 largest cities (ie, Seoul, Busan, and Daegu (HR, 1.62 [95% CI, 1.47-1.79] per IQR increase in OALAN) (eTable 7 in Supplement 1). However, in rural areas, no association was found between OALAN and risk of EAMD (*P* = .77) (Figure 2). An increased IQR (55.8 nW/cm^2^/sr) in OALAN showed an HR of 1.01 (95% CI, 0.84-1.22) for incident EAMD.

### Stratified Analysis and Sensitivity Analysis

The association between OALAN and EAMD risk was present in relatively older individuals, men, those with higher BMI (≥25.0), smokers, those who consumed alcohol, urban residents, and those with hypertension or dyslipidemia (Table 2 and eTable 8 in Supplement 1). To address potential confounding factors such as depression and sleep disorders, we performed a sensitivity analysis by excluding 23 674 participants with these conditions. The analysis showed that the estimated association between OALAN and risk of EAMD remained unchanged (eTable 9 in Supplement 1).

**Table 2.** Stratified Analyses of HRs for Incident Exudative Age-Related Macular Degeneration by Personal Characteristics at Baseline

Characteristic	Adjusted model, HR (95% CI)^a^	Effect modification
Age, y		
<70	1.20 (1.12-1.30)	1 [Reference]
≥70	1.42 (1.29-1.57)	<.001
Sex		
Women	1.99 (1.84-2.15)	1 [Reference]
Men	2.14 (1.91-2.38)	<.001
BMI		
<23.0	1.77 (1.62-1.93)	1 [Reference]
23.0-24.9	1.73 (1.54-1.95)	.06
≥25.0	2.00 (1.78-2.25)	.02
Smoking status		
Never	1.51 (1.40-1.62)	1 [Reference]
Ever	2.15 (1.94-2.39)	<.001
Alcohol consumption		
No	1.41 (1.27-1.57)	1 [Reference]
Yes	1.59 (1.48-1.71)	.004
Hypertension		
No	1.49 (1.36-1.65)	1 [Reference]
Yes	1.54 (1.36-1.73)	.002
Diabetes		
No	1.66 (1.55-1.77)	1 [Reference]
Yes	1.84 (1.64-2.06)	.32
Dyslipidemia		
No	1.71 (1.60-1.83)	1 [Reference]
Yes	1.72 (1.53-1.94)	.02
CKD		
No	1.67 (1.56-1.78)	1 [Reference]
Yes	1.83 (1.47-2.27)	.60
COPD		
No	1.66 (1.55-1.78)	1 [Reference]
Yes	1.84 (1.66-2.04)	.41
Cancer		
No	1.65 (1.55-1.77)	1 [Reference]
Yes	2.02 (1.73-2.35)	.02
Low income		
No	1.63 (1.55-1.77)	1 [Reference]
Yes	1.53 (1.36-1.72)	.08
Urban		
No	0.88 (0.74-1.05)	1 [Reference]
Yes	1.56 (1.42-1.72)	<.001
Low physical activity		
No	1.73 (1.59-1.88)	1 [Reference]
Yes	1.86 (1.68-2.07)	.22

Abbreviations: HR, hazard ratio; BMI, body mass index (calculated as weight in kilograms divided by height in meters squared); CKD, chronic kidney disease; COPD, chronic obstructive pulmonary disease.

^a^ Models were adjusted for all but the relevant characteristic among the following factors: age; sex; BMI; alcohol consumption; exercise status; income level; comorbidities, including hypertension, diabetes, dyslipidemia, CKD, COPD, and cancer; particulate matter with aerodynamic diameter of 10 μm or less; and nighttime traffic noise at the residential address. A linear relationship between outdoor artificial light at night and exudative age-related macular degeneration was assumed for this analysis.

## Discussion

In this nationwide population-based case-control study, OALAN at the residential address was associated with higher risk of incident EAMD, even after adjustment for a wide range of individual- and area-level risk factors. Notably, we observed regional disparities in this association. A significant association between OALAN and higher risk of incident EAMD was found solely in urban areas, where the mean OALAN level was 3 times higher than in rural areas. These regional differences suggest the presence of a potential threshold above which OALAN levels may increase the risk of EAMD. The OALAN levels in rural areas may not have surpassed the threshold associated with an increased risk. Additionally, the potential impact of region-specific factors cannot be entirely excluded. Despite our consideration of potential confounding factors, there remains a possibility that unaccounted variables, such as diet or lifestyle patterns, could have influenced the results in the differences between rural and urban areas.

We also found an association between OALAN and EAMD risk in elderly individuals (70 years or older), men, those living in an urban area, those with higher BMI (≥25.0), smokers, those who consumed alcohol, and those with hypertension or dyslipidemia. Our results are broadly consistent with insights provided by previous studies. Increasing age and cigarette smoking have been consistently identified as the main risk factors for EAMD.^3^ Although the findings remain inconclusive, various studies have reported possible associations of greater EAMD risk with higher BMI,^34,35^ alcohol consumption,^36^ hypertension,^37,38,39^ and dyslipidemia.^40,41^ Unhealthy lifestyle factors resulting in inflammation may be a causality shared by EAMD and cardiovascular diseases.^42^ Further investigation is warranted to elucidate the role of OALAN in the development and/or progression of EAMD, including its interaction with other known risk factors.

Although the pathogenesis of EAMD is not fully understood, light-induced damage to retinal pigment epithelium (RPE) cells has been extensively studied as an environmental risk factor.^43,44^ The impairment of RPE cells, which play essential roles in metabolic and supportive functions such as oxygen supply and waste removal from the retina, represents a crucial mechanism in the development and progression of EAMD.^45,46^ Whereas the epidemiological evidence on the association between sunlight exposure and EAMD has been inconclusive,^47,48,49^ the experimental evidence seems sufficient. A loss of viability has been observed in RPE cells after irradiation by either UV-A or UV-B.^50,51^ Excessive light exposure, particularly long-term blue light exposure, has been shown to disrupt mitochondrial dynamics, promote fragmentation, and cause oxidative stress, ultimately decreasing the viability of RPE cells by nearly 40%.^44,52^ In this context, it is conceivable that light pollution may cause dysfunction in RPE cells and affect progression to EAMD.

Circadian rhythms are central to the health effects of ALAN.^24^ In vertebrates, phagocytosis of the photoreceptor’s outer segments is synchronized by circadian rhythms.^53^ Impairment in the timely clearance of a phagocytosed photoreceptor’s outer segments can lead to cumulative oxidative damage in the RPE. In addition, dysregulation of the circadian clock by constant light exposure in animals has been found to enhance the process of angiogenesis.^54^

Hormone secretions, including melatonin, follow the circadian rhythm and play a significant role in modulating immune system activity.^24^ ALAN has been shown to inhibit melatonin secretion, leading to impaired immune function. Disruption of circadian rhythms can also trigger the production of proinflammatory cytokines and increase basal inflammation, independent of immune challenges.^55^ Considering the relationship between systemic inflammation, aging, and the immune system’s involvement in the development of EAMD,^42^ it is plausible that the association between OALAN and EAMD risk can be explained in terms of similar mechanisms, in addition to any direct effects on RPE cells. In fact, previous studies have suggested that melatonin plays a role in the progression of EAMD.^56,57,58^

Numerous natural protective mechanisms exist to safeguard the retina against harmful light rays. Notably, the crystalline lens plays a crucial role in filtering light, particularly blue light.^59^ Therefore, the presence and severity of cataracts or a history of cataract surgery may influence OALAN exposure and its potential correlation with AMD risk. Even UV- and blue-filtering intraocular lenses, commonly used after cataract surgery, may not provide sufficient photoprotective effects.^60^ In our analysis, due to limitations in the available claims data, verifying clinical records regarding the extent of cataracts was not feasible. Patients with cataracts severe enough to warrant surgical intervention may experience some level of protection against light exposure, but their patterns of light exposure may change after surgery. As a result, methodological constraints made it prohibitively challenging to comprehensively account for these cumulative effects.

Late-stage AMD can manifest in either exudative or nonexudative forms. A comprehensive meta-analysis conducted on a global scale revealed a higher prevalence of geographic atrophy, a subtype of nonexudative AMD, among White European relative to Asian individuals.^2^ This finding suggests the existence of distinct variations in pathophysiology and risk factors between the 2 types (ie, exudative and nonexudative) of AMD. The current analysis exclusively focused on the exudative form owing to the absence of adequate measures to ensure diagnostic accuracy for the nonexudative form in our data set. Further research is warranted to investigate whether light pollution is associated with progression to the nonexudative type of AMD.

### Limitations

This study has some limitations. First, OALAN derived from satellite data may not precisely capture individual ALAN exposure. Our study did not encompass an analysis of long-term OALAN fluctuations or an evaluation of temporal variations. Although we identified participants who had been living at the same residence during the study period, exposure misclassification remains possible if OALAN levels changed substantially in the same areas where people resided. Nonetheless, we believe that any measurement errors would likely appear as random deviations, not altering the core message of our study. Second, the current study is based on outdoor light at night, which is not necessarily equivalent to total light experienced since there may also be indoor sources of nighttime light as well or the use of light-blocking accessories or other adaptive behaviors that limit the amount of outdoor light penetrating a room. Hopefully, our work can inspire investigators of future studies to expand the scope of exposure assessment to include all types of light at night. Third, our estimates of OALAN were limited to the measurement of light intensity and did not account for variations in light wavelengths, which could be an important distinction. Shorter, blue-spectrum wavelength light is known to be more detrimental to sleep,^61,62^ which could be an issue as cities are moving toward shorter-wavelength light-emitting diode streetlights.^63^

## Conclusions

The findings of this case-control study suggest a link between higher levels of residential OALAN exposure and increased risk of incident EAMD. These findings align with the increasing body of evidence that emphasizes the negative impact of OALAN on health, further implicating OALAN as a potential risk factor for EAMD. Further studies incorporating comprehensive information on exposure, individual adaptive behaviors, and potential mediators are recommended to deepen our understanding in this area.

## Notes

Supplement 1. **eMethods.** Assessment of Air Pollution and Nighttime Noise **eFigure 1.** Flowchart of Enrollment of Study Population **eFigure 2.** Exposure-Response Curve for Association Between OALAN at Residential Address and Risk of Incident Exudative Age-Related Macular Degeneration **eTable 1.** Definitions of Covariates and Clinical Outcome **eTable 2.** Assessment of Proportional Hazard Assumption **eTable 3.** Summary of Multicollinearity Analysis **eTable 4.** Summary Statistics for the Participants' Baseline Characteristics by Quartile of Outdoor Artificial Light at Night **eTable 5.** Minimally Adjusted Regression Model Results Across Various Analyses **eTable 6.** Fully Adjusted Regression Model Results Across Various Analyses **eTable 7.** Association Between Outdoor Nighttime Light Exposure and the Risk of Exudative Age-Related Macular Degeneration in the 3 Largest Cities in South Korea **eTable 8.** Stratified Analyses of Hazard Ratios for Incident Exudative Age-Related Macular Degeneration by Personal Characteristics at Baseline **eTable 9.** Sensitivity Analysis of the Association Between Outdoor Nighttime Light Exposure and the Risk of Exudative Age-Related Macular Degeneration in the Cohort That Excludes Participants With Conditions That May Impair Sleep Quality
eReference

Supplement 2.
Data Sharing Statement
