# Supplementary material for: Nighttime Outdoor Artificial Light and Risk of Age-Related Macular Degeneration
Source: JAMA Netw Open. 2024 Jan 16;7(1):e2351650. doi: 10.1001/jamanetworkopen.2023.51650 (PMC10792474; doi:10.1001/jamanetworkopen.2023.51650)
Supplement: Supplement 1. — eMethods. Assessment of Air Pollution and Nighttime Noise eFigure 1. Flowchart of Enrollment of Study Population eFigure 2. Exposure-Response Curve for Association Between OALAN at Residential Address and Risk of Incident Exudative Age-Related Macular Degeneration eTable 1. Definitions of Covariates and Clinical Outcome eTable 2. Assessment of Proportional Hazard Assumption eTable 3. Summary of Multicollinearity Analysis eTable 4. Summary Statistics for the Participants' Baseline Characteristics by Quartile of Outdoor Artificial Light at Night eTable 5. Minimally Adjusted Regression Model Results Across Various Analyses eTable 6. Fully Adjusted Regression Model Results Across Various Analyses eTable 7. Association Between Outdoor Nighttime Light Exposure and the Risk of Exudative Age-Related Macular Degeneration in the 3 Largest Cities in South Korea eTable 8. Stratified Analyses of Hazard Ratios for Incident Exudative Age-Related Macular Degeneration by Personal Characteristics at Baseline eTable 9. Sensitivity Analysis of the Association Between Outdoor Nighttime Light Exposure and the Risk of Exudative Age-Related Macular Degeneration in the Cohort That Excludes Participants With Conditions That May Impair Sleep Quality eReference [file jamanetwopen-e2351650-s001.pdf]

Supplementary Online Content

Kim SH, Kim YK, Shin YI, et al. Nighttime outdoor artificial light and risk of age-related macular degeneration. *JAMA Netw Open*. 2024;6(1):e2351650. doi:10.1001/jamanetworkopen.2023.51650

eMethods. Assessment of Air Pollution and Nighttime Noise

eFigure 1. Flowchart of Enrollment of Study Population

eFigure 2. Exposure-Response Curve for Association Between OALAN at Residential Address and Risk of Incident Exudative Age-Related Macular Degeneration

eTable 1. Definitions of Covariates and Clinical Outcome

eTable 2. Assessment of Proportional Hazard Assumption

eTable 3. Summary of Multicollinearity Analysis

eTable 4. Summary Statistics for the Participants' Baseline Characteristics by Quartile of Outdoor Artificial Light at Night

eTable 5. Minimally Adjusted Regression Model Results Across Various Analyses

eTable 6. Fully Adjusted Regression Model Results Across Various Analyses

eTable 7. Association Between Outdoor Nighttime Light Exposure and the Risk of Exudative Age-Related Macular Degeneration in the 3 Largest Cities in South Korea

eTable 8. Stratified Analyses of Hazard Ratios for Incident Exudative Age-Related Macular Degeneration by Personal Characteristics at Baseline

eTable 9. Sensitivity Analysis of the Association Between Outdoor Nighttime Light Exposure and the Risk of Exudative Age-Related Macular Degeneration in the Cohort That Excludes Participants With Conditions That May Impair Sleep Quality

eReference.

This supplementary material has been provided by the authors to give readers additional information about their work.

© 2024 Kim SH et al. *JAMA Network Open*.

eMethods. Assessment of Air Pollution and Nighttime Noise

We estimated the annual concentration of particulate matter of aerodynamic diameter ≤ 10 μm (PM_{10}) in each participant's administrative district of residence using validated machine-learning-based models. The model was constructed by ensemble method entailing random forest regression, gradient boosting, and neural network. Additional methodological details are previously reported and available upon request (www.datascience4health.com).

To measure night-time noise levels, we obtained data from the National Noise Information System (https://www.noiseinfo.or.kr/). Average yearly district-level noise was used as a proxy for individual exposure.

© 2024 Kim SH et al. *JAMA Network Open*.

eFigure 1. Flowchart of Enrollment of Study Population

EAMD = exudative age-related macular degeneration; PM_{10} = particulate matter with aerodynamic diameter ≤ 10 μm

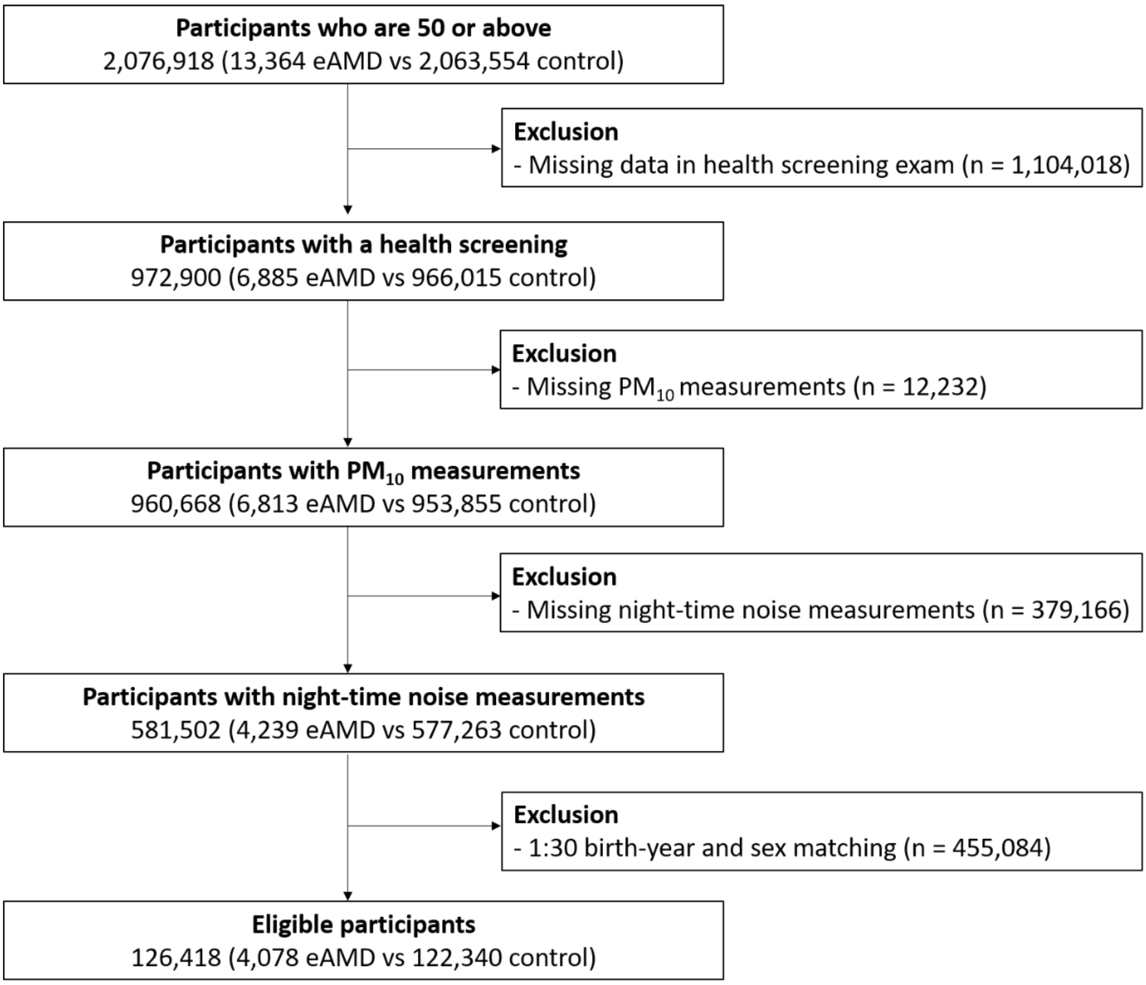

© 2024 Kim SH et al. *JAMA Network Open*.

eFigure 2. Exposure-Response Curve for Association Between OALAN at Residential Address and Risk of Incident Exudative Age-Related Macular Degeneration

OALAN was fitted as a smooth term using a penalized smoothing spline with four degrees of freedom. The solid line represents urban areas, the dashed line represents rural areas' outcomes. The shaded area represents the 95% confidence interval. HR = hazard ratio.

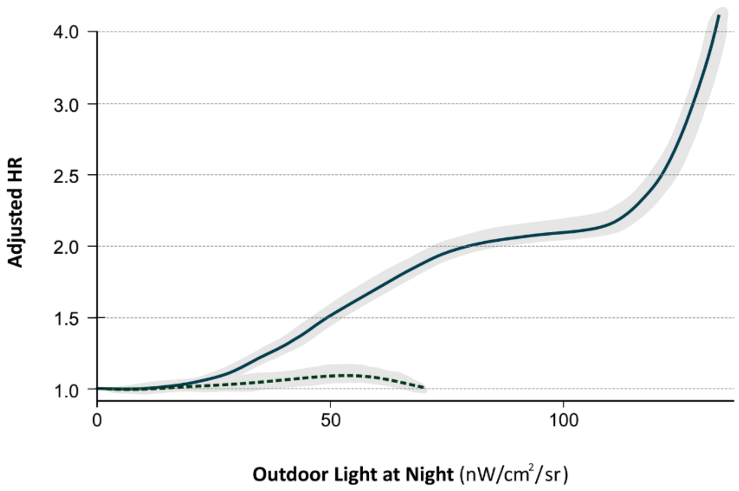

© 2024 Kim SH et al. *JAMA Network Open*.

eTable 1. Definitions of Covariates and Clinical Outcome

Diagnosis	ICD-10 code and definition	Diagnostic definition
**Inclusion criteria**		
**Exudative AMD**	V201 (identified using the registration program database for rare intractable diseases)	Outpatient department≥2
**Comorbidities**		
**Hypertension**	I10-I13, I15; and minimum 1 prescription of anti-hypertensive drug (thiazide, loop diuretics, aldosterone antagonist, alpha-/beta-blocker, calcium-channel blocker, angiotensin-converting enzyme inhibitor, angiotensin II receptor blocker).	Admission≥1 or outpatient department≥2
	or systolic/diastolic blood pressure ≥ 130/80 mmHg	Based on the results of the health exam
**Diabetes mellitus**	E11-E14; and minimum 1 prescription of anti-diabetic drugs (sulfonylureas, metformin, meglitinides, thiazolidinediones, dipeptidyl peptidase-4 inhibitors, α-glucosidase inhibitors, and insulin).	Admission≥1 or outpatient department≥2
	or fasting glucose level ≥ 126 mg/dL	Based on the results of the health exam
**Dyslipidemia**	E78	Admission or outpatient department≥1
	or Total cholesterol ≥ 240 mg/dL	Based on the results of the health exam
**CKD**	eGFR<60ml/min/1.73m^2	Based on the results of the health exam
**COPD**	J41-44	Admission or outpatient department≥1
**Cancer**	C00-97 and rare intractable diseases code (V193)	Admission or outpatient department≥1
**Health exam questionnaire**		
**Alcohol consumption (daily alcohol intake amount)**	Non (0 g)<br>Mild (0 < g <10)<br>Moderate (10 ≤ g < 20)<br>Heavy (20 ≤ g < 40)<br>Extremely heavy (≥ 40g)	Based on the results of the health exam
**Regular exercise**	Light (0 days per week or METs < 600)<br>Moderate (1-4 days per week or 600 ≤ METs < 3000)<br>Vigorous (5 or more days per week or METs ≥ 3000)	Based on the results of the health exam
**Low income**	Income lowest 20% and medical aid	

ICD, International Classification of Diseases; AMD, age-related macular degeneration; DR, diabetic retinopathy; CKD, chronic kidney disease; COPD, chronic obstructive pulmonary disease; eGFR, estimated glomerular filtration rate; MET, metabolic equivalent of task.

© 2024 Kim SH et al. *JAMA Network Open*.

eTable 2. Assessment of Proportional Hazard Assumption

Variable	Chi-square	P value
OALAN	0.33	0.56
Age above 70	0.00	0.99
Sex	1.15	0.28
BMI	0.06	0.80
Hypertension	0.04	0.85
Diabetes mellitus	7.49	0.01
Dyslipidemia	0.74	0.39
COPD	1.62	0.20
Chronic kidney disease	0.00	0.95
Cancer	0.01	0.94
Smoking status	4.67	0.10
Alcohol consumption	2.14	0.71
Physical activity level	1.69	0.43
Income level	6.03	0.11
Urban	0.09	0.76
PM_{10}	0.87	0.35
Night-time outdoor noise	0.57	0.45
Global	30.5	0.21

OALAN, outdoor artificial light at night; BMI, body mass index; COPD, chronic obstructive pulmonary disease; PM_{10}, particulate matter with aerodynamic diameter ≤ 10 μm

Schoenfeld residual plot analysis for diabetes

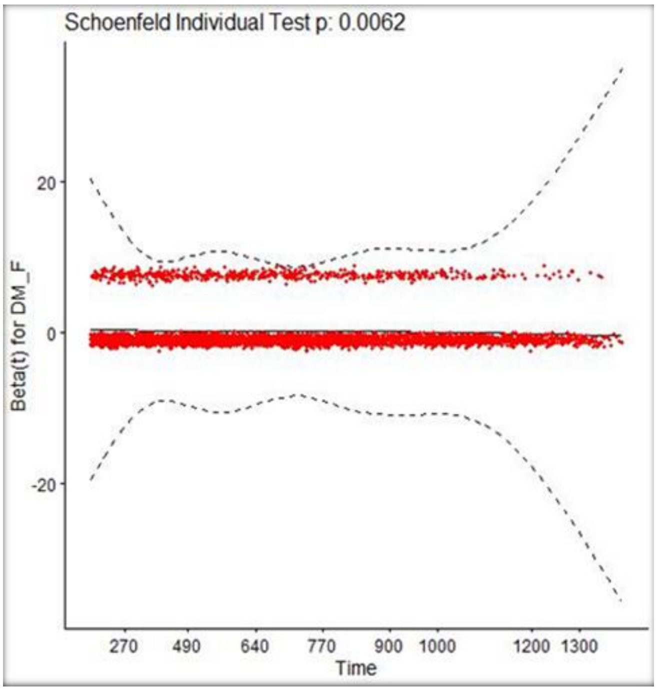

© 2024 Kim SH et al. *JAMA Network Open*.

eTable 3. Summary of Multicollinearity Analysis

Variable	$GVIF^{(0.5*df)}$
OALAN	1.47
Age above 70	1.01
Sex	1.30
BMI	1.46
Hypertension	1.05
Diabetes Mellitus	1.01
Dyslipidemia	1.01
COPD	1.01
Chronic kidney disease	1.01
Cancer	1.01
Smoking Status	1.12
Alcohol Consumption	1.04
Physical Activity Level	1.01
Income Level	1.01
Urban	1.12
PM_{10}	1.04
Night-time outdoor noise	1.24

GVIF, Generalized variance inflation factor; OALAN, outdoor artificial light at night; BMI, body mass index; COPD, chronic obstructive pulmonary disease; PM_{10}, particulate matter with aerodynamic diameter ≤ 10 μm.
$GVIF^{(0.5*df)}$ > 2 indicates presence of multicollinearity.

© 2024 Kim SH et al. *JAMA Network Open*.

eTable 4. Summary Statistics for the Participants' Baseline Characteristics by Quartile of Outdoor Artificial Light at Night

Characteristics	Quartile 1 (*n* = 31,253)	Quartile 2 (*n* = 32,185)	Quartile 3 (*n* = 30,898)	Quartile 4 (*n* = 32,082)
Light at night (nW/cm^2/sr)	8.1 ± 3.3	25.4 ± 8.2	47.2 ± 9.1	92.7 ± 20.0
PM_{10} (mg/m^3)	51.8 ± 5.2	50.9 ± 5.4	53.9 ± 4.7	55.1 ± 3.6
Night-time traffic noise (dB)	54.4 ± 2.6	53.7 ± 2.3	56.7 ± 4.0	56.7 ± 3.3
Age, years	69.6 ± 6.4	67.0 ± 7.9	64.6 ± 8.2	62.9 ± 7.4
Male sex	18,070 (57.8)	21,246 (66.0)	21,208 (68.6)	17,720 (55.2)
BMI (kg/m^2)	23.9 ± 3.2	24.0 ± 3.0	24.0 ± 2.9	24.1 ±2.9
Comorbidities				
Hypertension	23,587 (75.5)	23,168 (72.0)	21,203 (68.6)	21,527 (67.1)
Diabetes mellitus	3,727 (11.9)	4,056 (12.6)	3,830 (12.4)	3,763 (11.7)
Dyslipidemia	3,809 (12.2)	3,917 (12.2)	3,938 (12.8)	4,378 (13.7)
COPD	5,727 (18.3)	5,895 (18.3)	4,696 (15.2)	4,595 (14.3)
CKD	756 (2.4)	701 (2.2)	572 (1.9)	581 (1.8)
Cancer	1,424 (4.6)	1,487 (4.6)	1,304 (4.2)	1,165 (3.6)
Smoking status				
Never smokers	21,711 (69.5)	20,518 (63.8)	18,530 (60.0)	21,613 (67.4)
Former smokers	4,472 (14.3)	6,067 (18.9)	6,179 (20.0)	5,326 (16.6)
Current smokers	5,070 (16.2)	5,600 (17.4)	6,189 (20.0)	5,143 (16.0)
Alcohol drinking				
Non drinker	21,567 (69.0)	20,429 (63.5)	18,388 (59.5)	20,515 (64.0)
Mild drinker	4,336 (13.9)	5,619 (17.5)	5,777 (18.7)	5,635 (17.6)
Moderate drinker	2,077 (6.7)	2,842 (8.8)	3,060 (9.9)	2,856 (8.9)
Heavy drinker	1,949 (6.2)	2,144 (6.7)	2,351 (7.6)	2,073 (6.5)
Extremely Heavy drinker	1,324 (4.2)	1,151 (3.6)	1,322 (4.3)	1,003 (3.1)
Exercise (physical activity)				
Light activity	17,073 (54.6)	13,422 (41.7)	11,747 (38.0)	12,933 (40.3)
Moderate activity	10,167 (32.5)	13,624 (42.3)	14,143 (45.8)	14,541 (45.3)
Vigorous activity	4,013 (12.8)	5,139 (16.0)	5,008 (16.2)	4,608 (14.4)
Household income percentiles				
< 5%	5,383 (17.2)	5,792 (18.0)	5,710 (18.5)	6,273 (19.6)
6-10%	4,862 (15.6)	4,841 (15.0)	5,128 (16.6)	5,536 (17.3)
11-15%	7,263 (23.2)	7,264 (22.6)	7,377 (23.9)	7,702 (24.0)
16-20%	13,745 (44.0)	14,288 (44.4)	12,683 (41.1)	12,571 (39.2)

Continuous variables are presented as mean ± standard deviation, and categorical variables are presented as number (percentage).
PM_{10}, particulate matter with aerodynamic diameter ≤ 10 μm; BMI, body mass index; COPD, chronic obstructive pulmonary disease; CKD, chronic kidney disease.

© 2024 Kim SH et al. *JAMA Network Open*.

eTable 5. Minimally Adjusted Regression Model Results Across Various Analyses

Analysis	Variable	HR (95% CI)
HR per OALAN IQR increase with linearity assumption	Age	1.33 (1.24, 1.43)
	Sex	1.01 (0.94, 1.08)
HRs for top three OALAN Q *vs.* lowest Q	Age	1.35 (1.26, 1.45)
	Sex	0.97 (0.91, 1.04)
HR curve with a penalized smoothing spline	Age	1.17 (1.10, 1.26)
	Sex	0.92 (0.86, 0.98)

HR, hazard ratio; CI, confidence interval; OALAN, outdoor artificial light at night; IQR, interquartile range; Q, quartile

© 2024 Kim SH et al. *JAMA Network Open*.

eTable 6. Fully Adjusted Regression Model Results Across Various Analyses

Variable	HR per OALAN IQR increase with linearity assumption	HRs for Top three OALAN Q *vs.* lowest Q	HR curve with a penalized smoothing spline
Age	1.08 (1.01-1.16)	1.10 (1.02-1.18)	1.11 (1.03-1.19)
Sex	1.13 (1.04-1.23)	1.09 (1.00-1.18)	1.03 (0.95-1.12)
BMI	1.02 (1.01-1.03)	1.02 (1.01-1.03)	1.02 (1.01-1.03)
Hypertension	1.03 (0.96-1.10)	1.03 (0.96-1.11)	1.02 (0.95-1.10)
Diabetes mellitus	1.10 (1.01-1.21)	1.11 (1.01-1.22)	1.11 (1.01-1.22)
Dyslipidemia	1.01 (0.92-1.11)	1.01 (0.92-1.11)	1.01 (0.92-1.11)
COPD	1.11 (1.03-1.21)	1.11 (1.02-1.20)	1.11 (1.02-1.20)
Chronic kidney disease	1.10 (0.90-1.35)	1.11 (0.90-1.36)	1.11 (0.90-1.36)
Cancer	1.22 (1.06-1.40)	1.11 (1.02-1.20)	1.20 (1.04-1.38)
Smoking status			
Never smokers	*Reference*	*Reference*	*Reference*
Former smokers	1.50 (1.37-1.65)	1.50 (1.37-1.65)	1.51 (1.31-1.65)
Current smokers	1.31 (1.19-1.45)	1.31 (1.19-1.45)	1.31 (1.19-1.44)
Alcohol consumption			
Non drinker	*Reference*	*Reference*	*Reference*
Mild drinker	0.92 (0.84-1.01)	0.92 (0.85-1.01)	0.93 (0.85-1.02)
Moderate drinker	0.85 (0.75-0.96)	0.85 (0.75-0.96)	0.85 (0.75-0.96)
Heavy drinker	0.85 (0.74-0.97)	0.85 (0.74-0.97)	0.85 (0.74-0.97)
Extremely Heavy drinker	0.83 (0.70-0.99)	0.83 (0.70-0.99)	0.84 (0.70-1.00)
Physical activity			
Light activity	*Reference*	*Reference*	*Reference*
Moderate activity	1.09 (1.02-1.17)	1.08 (1.01-1.16)	1.09 (1.02-1.17)
Vigorous activity	1.05 (0.96-1.16)	1.04 (0.95-1.15)	1.04 (0.95-1.14)
Income level			
Quartile 1	*Reference*	*Reference*	*Reference*
Quartile 2	0.98 (0.88-1.10)	0.99 (0.88-1.10)	0.99 (0.89-1.10)
Quartile 3	0.99 (0.90-1.09)	0.99 (0.90-1.09)	0.99 (0.90-1.09)
Quartile 4	1.15 (1.05-1.26)	1.15 (1.05-1.25)	1.15 (1.05-1.25)
Residential area			
Rural	*Reference*	*Reference*	*Reference*
Urban	1.09 (0.98-1.22)	1.05 (0.95-1.15)	1.19 (1.07-1.33)
PM_{10}	0.98 (0.97-0.98)	0.97 (0.97-0.98)	0.98 (0.97-0.99)
Night-time outdoor noise	1.00 (0.99-1.01)	1.00 (0.99-1.01)	0.99 (0.98-1.00)

Presented as HR (95% CI)
HR, hazard ratio; CI, confidence interval; OALAN, outdoor artificial light at night; IQR, interquartile range; Q, quartile; BMI, body mass index; COPD, chronic obstructive pulmonary disease; PM_{10}, particulate matter with aerodynamic diameter ≤ 10 μm

© 2024 Kim SH et al. *JAMA Network Open*.

eTable 7. Association Between Outdoor Nighttime Light Exposure and the Risk of Exudative Age-Related Macular Degeneration in the 3 Largest Cities in South Korea

	**Number of events**	**Minimally adjusted model*** **HR (95% CI)**	**Fully adjusted model†** **HR (95% CI)**
Incident EAMD in Seoul, Busan, and Daegu (the three largest cities)			
Quartile 1 (median 44.4 nW/cm^2/sr)	380	*Reference*	*Reference*
Quartile 2 (median 65.0 nW/cm^2/sr)	447	1.27 (1.11, 1.46)	1.21 (1.05, 1.39)
Quartile 3 (median 89.6 nW/cm^2/sr)	499	1.61 (1.40, 1.84)	1.43 (1.23, 1.67)
Quartile 4 (median 121.2 nW/cm^2/sr)	581	1.92 (1.68, 2.20)	1.67 (1.42, 1.96)
P for trend‡		0.003	0.002
Continuous light at night (per IQR increase§)		1.71 (1.57, 1.85)	1.62 (1.47, 1.79)

HR, hazard ratio; CI, confidence interval; EAMD, exudative age-related macular degeneration; IQR, interquartile range.
*Models included age and sex.
†Models were additionally adjusted for baseline body mass index, drinking status, exercise status, income level, comorbidities including hypertension, diabetes mellitus, dyslipidamia, chronic kidney disease, chronic obstructive pulmonary disease, cancer, fine particulate matter (PM_{10}), and night-time traffic noise at the residential address.
‡Test for trend is based on the median value for each quartile.
§An IQR increase in outdoor light at night at the residential address is 55.8 nW/cm^2/sr.

© 2024 Kim SH et al. *JAMA Network Open*.

eTable 8. Stratified Analyses of Hazard Ratios for Incident Exudative Age-Related Macular Degeneration by Personal Characteristics at Baseline

Characteristics	Subgroup	Effect modification for quartile analysis	Effect modification for spline analysis
Age	< 70 y	*Reference*	*Reference*
	≥70 y	< 0.001	< 0.001
Sex	Male	*Reference*	*Reference*
	Female	< 0.001	< 0.001
BMI	< 23.0 kg/m^2	*Reference*	*Reference*
	23.0–24.9 kg/m^2<br>≥ 25.0 kg/m^2	< 0.001	0.126
Smoking status	Never smoker	*Reference*	*Reference*
	Ever smoker	< 0.001	< 0.001
Alcohol consumption	No	*Reference*	*Reference*
	Yes	< 0.001	< 0.001
Hypertension	No	*Reference*	*Reference*
	Yes	< 0.001	0.002
Diabetes mellitus	No	*Reference*	*Reference*
	Yes	0.098	0.964
Dyslipidemia	No	*Reference*	*Reference*
	Yes	0.006	0.405
CKD	No	*Reference*	*Reference*
	Yes	0.765	0.250
COPD	No	*Reference*	*Reference*
	Yes	< 0.001	0.073
Cancer	No	*Reference*	*Reference*
	Yes	0.052	0.103
Low Income	No	*Reference*	*Reference*
	Yes	0.013	0.13
Urban	No	*Reference*	*Reference*
	Yes	< 0.001	< 0.001
Low Physical Activity	No	*Reference*	*Reference*
	Yes	< 0.001	0.004

HR, hazard ratio; CI, confidence interval; BMI, body mass index; CKD, chronic kidney disease; COPD; chronic obstructive pulmonary disease.

*Models were adjusted for all but the relevant characteristic among the following factors: age, sex, BMI, drinking status, exercise status, income level, comorbidities including hypertension, diabetes mellitus, dyslipidemia, CKD, COPD, cancer, fine particulate matter (PM_{10}), and night-time traffic noise at the residential address.

© 2024 Kim SH et al. *JAMA Network Open*.

eTable 9. Sensitivity Analysis of the Association Between Outdoor Nighttime Light Exposure and the Risk of Exudative Age-Related Macular Degeneration in the Cohort That Excludes Participants With Conditions That May Impair Sleep Quality

	**Minimally adjusted model*<br>HR (95% CI)**	**Fully adjusted model†<br>HR (95% CI)**
Incident EAMD		
Quartile 1 (median 8.9 nW/cm^2/sr)	*Reference*	*Reference*
Quartile 2 (median 29.2 nW/cm^2/sr)	0.96 (0.86, 1.07)	0.94 (0.84, 1.05)
Quartile 3 (median 48.4 nW/cm^2/sr)	1.22 (1.10, 1.36)	1.33 (1.19, 1.48)
Quartile 4 (median 93.5 nW/cm^2/sr)	1.76 (1.59, 1.94)	1.96 (1.76, 2.18)
P for trend‡	0.042	0.041
Continuous light at night (per IQR increase§)	1.53 (1.45, 1.61)	1.65 (1.56, 1.75)
Incident EAMD in urban area		
Quartile 1 (median 22.1 nW/cm^2/sr)	*Reference*	*Reference*
Quartile 2 (median 44.2 nW/cm^2/sr)	1.55 (1.35, 1.77)	1.69 (1.46, 1.96)
Quartile 3 (median 72.8 nW/cm^2/sr)	1.84 (1.62, 2.10)	2.04 (1.75, 2.38)
Quartile 4 (median 97.4 nW/cm^2/sr)	2.05 (1.80, 2.33)	2.31 (1.97, 2.70)
P for trend‡	0.033	0.033
Continuous light at night (per IQR increase§)	1.60 (1.50, 1.72)	1.72 (1.58, 1.86)
Incident EAMD in rular area		
Quartile 1 (median 3.5 nW/cm^2/sr)	*Reference*	*Reference*
Quartile 2 (median 9.3 nW/cm^2/sr)	0.76 (0.64, 0.92)	0.94 (0.77, 1.13)
Quartile 3 (median 16.2 nW/cm^2/sr)	0.89 (0.75, 1.06)	1.13 (0.93, 1.37)
Quartile 4 (median 47.4 nW/cm^2/sr)	0.76 (0.64, 0.91)	1.06 (0.84, 1.34)
P for trend‡	0.394	0.601
Continuous light at night (per IQR increase§)	0.80 (0.66, 0.97)	1.00 (0.78, 1.27)

HR, hazard ratio; CI, confidence interval; EAMD, exudative age-related macular degeneration; IQR, interquartile range.
*Models included age and sex.
†Models were additionally adjusted for baseline body mass index, drinking status, exercise status, income level, comorbidities including hypertension, diabetes mellitus, dyslipidemia, chronic kidney disease, chronic obstructive pulmonary disease, cancer, fine particulate matter (PM10), and night-time traffic noise at the residential address.
‡Test for trend is based on the median value for each quartile.
§An IQR increase in outdoor light at night at the residential address is 55.8 nW/cm^2/sr.

eReference.

Park J, Kang C, Min J, et al. Association of long-term exposure to air pollution with chronic sleep deprivation in South Korea: A community-level longitudinal study, 2008–2018. Environ Res. 2023;228:115812.

© 2024 Kim SH et al. *JAMA Network Open*.
