# Supplementary material for: Nighttime Outdoor Artificial Light and Risk of Age-Related Macular Degeneration
Source: JAMA Netw Open. 2024 Jan 16;7(1):e2351650. doi: 10.1001/jamanetworkopen.2023.51650 (PMC10792474; doi:10.1001/jamanetworkopen.2023.51650)
Supplement: Supplement 2. — Data Sharing Statement [file jamanetwopen-e2351650-s002.pdf]

Data Sharing Statement

Kim. Nighttime Outdoor Artificial Light and Risk of Age-Related Macular Degeneration. *JAMA Netw Open*. Published January 16, 2024. doi:10.1001/jamanetworkopen.2023.51650

Data
Data available: Yes
Data types: Deidentified participant data
How to access data: All data analyzed during the sustained study are available at the National Health Insurance Data Sharing Service (accessed at https://nhiss.nhis.or.kr/bd/ab/bdaba000eng.do) after appropriate review and payment of a fee.
When available: With publication

Supporting Documents
Document types: Statistical/analytic code
How to access documents: Supporting information will be available with reasonable request to the corresponding authors.
When available: With publication

Additional Information
Who can access the data: Researchers whose proposed use of the data has been approved
Types of analyses: For academic purpose
Mechanisms of data availability: after approval of a proposal
